# 3D Heads-Up Display vs. Standard Operating Microscope Vitrectomy for Rhegmatogenous Retinal Detachment

**DOI:** 10.3389/fmed.2020.615515

**Published:** 2020-12-22

**Authors:** Ben Asani, Jakob Siedlecki, Benedikt Schworm, Wolfgang J. Mayer, Thomas C. Kreutzer, Nikolaus Luft, Siegfried G. Priglinger

**Affiliations:** Department of Ophthalmology, Ludwig-Maximilians-University, Munich, Germany

**Keywords:** rhegmatogenous retinal detachments, 3D heads-up display, NGENUITY, vitrectomy, operating microscope

## Abstract

**Purpose:** To assess the efficacy and outcomes of 23-gauge vitreoretinal surgery for rhegmatogenous retinal detachment using a three-dimensional heads-up display (3D HUD) surgical platform as compared to a standard operating microscope (SOM) setting.

**Design:** Retrospective cohort study.

**Participants:** One hundred and forty consecutive eyes of 140 patients with primary retinal detachment.

**Methods:** All eyes underwent 23-gauge pars plana vitrectomy for primary retinal detachment using either a 3D HUD (NGENUITY; Alcon Inc., Fort Worth, Texas, USA; *n* = 70 eyes) or a SOM setting (*n* = 70 eyes); in cases of significant cataract, additional phacoemulsification with intraocular lens (IOL) implantation was performed. Minimum follow-up was 2 months.

**Main Outcome Measures:** Primary retinal reattachment rate, rate of proliferative vitreoretinopathy (PVR), best-corrected visual acuity (BCVA), and duration of surgery.

**Results:** There were 70 eyes each in the 3D HUD and the SOM group. Both groups did not differ concerning age (*p* = 0.70), extent of retinal detachment (*p* = 0.07), number of retinal tears (*p* = 0.40), macular involvement (*p* = 0.99), and preoperative BCVA (*p* = 0.99). Postoperatively, 3D HUD and SOM were comparable concerning the primary retinal reattachment rate (88.6 vs. 94.3%; *p* = 0.37), the development of postoperative PVR (12.9% vs. 7.1%; *p* = 0.40) and final BCVA (0.26 ± 0.40 vs. 0.21 ± 0.38 logMAR; *p* = 0.99). Duration of surgery was significantly longer in the 3D HUD group (66.2 ± 16.5 vs. 61.2 ± 17.1 min; *p* = 0.04), an effect which however vanished after a “learning curve” of the first 35 eyes (*p* = 0.49).

**Conclusions:** On par results to a conventional operating microscope can be achieved with a 3D HUD setting when performing 23-gauge vitreoretinal surgery for rhegmatogenous retinal detachment, including the primary retinal reattachment rate, the incidence of postoperative PVR and final BCVA. However, duration of surgery might initially be slightly longer with 3D HUD, suggesting the effect of a learning curve.

## Introduction

Despite major technological advancements in the medical field, there has been limited innovation in digital assisted ophthalmic surgery in the last decade. Latest developments include intraoperative optical coherence tomography (OCT) (1, 2), which by now has not found its way into standard clinical practice. Besides intraoperative tracking for toric intraocular lens alignment displayed via a heads-up display (HUD) in one ocular (3), there has been none but minor digital amendments to the standard operating microscope (SOM).

The recent release of a three-dimensional heads-up display (3D HUD; NGENUITY; Alcon Inc., Fort Worth, Texas, USA) pursues an essentially different approach to visualize intraocular surgery by replacing the traditional oculars with a digital screen. Hence, performing surgery with a 3D HUD can literally be performed “heads-up” with polarizing spectacles enabling a stereoscopic view of the surgical field that is captured via a 3D stereoscopic, high-definition digital video camera and displayed on a high-definition monitor. According to the manufacturer, the 3D HUD is supposed to support surgeons with improved magnification, a wider depth of field and a larger visual field as compared with contemporary SOM systems. Further advantages pertain to the applicability of instant digital filters (e.g., to enhance vitreous visability) as well as the technology's extensive teaching possibilities as the surgeon's view is identical with the audience's (e.g., fellows, nurses, and students).

As of today, different studies have evaluated the safety and efficacy of a 3D HUD setting for different types of ophthalmic surgical procedures, including cataract (4) or macular surgery (5).

In spite of these favorable initial reports, it is still to be scientifically determined whether the 3D HUD technology may be unconditionally employed for more complex posterior segment surgery such as pars plana vitrectomy for rhegmatogenous retinal detachment. (6) Hence, the aim of the present study was to evaluate the safety and efficacy of 3D HUD pars plana vitrectomy for primary rhegmatogenous retinal detachment. Additionally, we investigated if there was a learning curve present by partitioning the observed patients into 35 first and 35 last operated cases for each group.

## Methods

This retrospective case series included patients that underwent 23-gauge three-port pars plana vitrectomy for primary retinal detachment between July 1st 2017 and January 31st 2020 at the University Eye Hospital of the Ludwig-Maximilians University (Munich, Germany). We collected an equal amount of consecutive cases of primary retinal detachment before and after the implementation of a 3D HUD system (NGENUITY, Alcon Inc., Fort Worth, TX) mounted on a SOM (OPMI Lumera 700 with ReSight; Carl Zeiss Meditec AG; Jena, Germany) in our institution on July 1st 2018. All patients operated before that date underwent surgery with SOM visualization (OPMI Lumera 700 with ReSight) and all patients operated afterwards underwent 3D HUD surgery. “Primary” retinal detachment was defined as: No previous retinal surgery, no preoperative proliferative vitreoretinopathy (PVR) (Grade B and more severe) that requires the peeling of the internal limiting membrane, and no trauma. Additional exclusion criteria included anterior or posterior segment comorbidities that could influence the surgical procedure or affect functional outcomes (e.g., uncontrolled glaucoma, full-thickness macular hole). The study was approved by the institutional review board of our institution and adhered to the tenets of the Declaration of Helsinki. Written informed consent was obtained from each participant prior to the intervention and all testing outlined herein. The total number of patients was chosen by setting the power = 0.8, *a* = 0.05 and the inferiority limit = 0.06.

### Vitrectomy Surgery

All patients underwent standard 23-gauge three-port pars plana vitrectomy either under retrobulbar block or general anesthesia. Surgery was performed by a single experienced ophthalmic surgeon (S.G.P.) in all cases. In case of significant co-existent crystalline lens opacification according to the surgeon's judgment, simultaneous phacoemulsification with intraocular lens (IOL) implantation was performed at the beginning of the case. The surgical protocol for vitrectomy included the creation of three-port sclerotomies 3.5 mm from the limbus with two 23-gauge cannulas as working ports (for higher stability as well as a higher spectrum for the used instruments) and one 27-gauge cannula as a port for the infusion (to prevent excessive hypotony when removing the port), detachment of the anterior hyloid membrane, vitreous staining with triamcinolone, thorough vitrectomy, instillation of perfluorocarbon liquid (PFCL), shaving of the vitreous base with globe indentation (performed by the surgeon using a chandelier light), endolaser coagulation around the retinal tears and peripheral degenerative areas, fluid-air exchange, endotamponade with either gas (C2F6 gas tamponade: 16% Hexafluorethane, 84% air) or silicone oil (2,000 or 5,000 centistokes) according to the nature of the retinal detachment and lastly the suturing of sclerotomies. The operating time between insertion of the lid speculum and removal of the lid speculum as well as intraoperative complications were documented. All patients are routinely advised to maintain the face-down position immediately after vitrectomy for a couple of hours. Therafter, patients are asked to turn their face down toward the opposite site of the retinal tear.

### Preoperative and Postoperative Examinations

Clinical examinations included best-corrected visual acuity testing using standard Early Treatment Diabetic Retinopathy Study (ETDRS) chart at testing distance of 4 m, intraocular pressure measurements using Goldmann applanation tonometry, dilated indirect fundoscopy as well as spectral-domain optical coherence tomography of the macula (Spectralis; Heidelberg Engineering GmbH, Heidelberg, Germany). The metrics “counting fingers,” “hand movement,” and “light perception” were converted to 1.98, 2.28, and 2.80 logMAR, respectively, as previously reported by Lange et al. (7). Succesfull reattachment of the retina was defined as no incidence of retinal redetachment in the follow-up postoperative examinations. Proliferative vitreoretinopathy (PVR) was assessed through dilated fundoscopy and classified as Grade A, B, C according to the Retina Society Terminology Committee (8) and as updated by Machemer et al. (9).

For the purpose of this study, postoperative PVR was considered as a significant complication when it required surgical intervention (peeling of tractional membranes) and was therefore defined as Grade C. Attachment of the fovea was assessed as “macula on” (fovea still attached) or “macula off” (fovea detached) with dilated indirect fundoscopy. All study-related postoperative examinations were conducted a minimum of 2 months postoperatively. The postoperative complications we evaluated were macular edema, retinal re-detachment, endophthalmitis, hypotony, vitreous hemorrhage, and postoperative PVR. Other follow up visits were conducted by the patients' local ophthalmologists and only referred back to our institution in cases of complications. All cases mentioned in this study that suffered retinal re-detachment initially received a gas tamponade. In case of further necessary vitrectomy procedures, either gas or silicone oil depending on the severity and nature of the re-detachment was used according to the surgeon's judgement.

### Statistical Analysis

All statistical analysis was performed using R [R Core Team (2019). R: A language and environment for statistical computing. R Foundation for Statistical Computing, Vienna, Austria; http://www.R-project.org]. Normality of data was assessed using the Shapiro–Wilk Test. We applied the Mann–Whitney-*U* Test for group comparisons of non-parametric parameters (i.e., *X, Y, Z*), the independent samples *t*-test for parametric comparisons (i.e., age) and the Pearson's chi-squared test for binary comparisons (e.g., retinal re-detachment). The level of statistical significance was defined as *p* < 0.05.

## Results

The present analysis included a total of 140 eyes of 140 patients (Table 1). The 3D HUD group and the SOM group each comprised 70 eyes of 70 patients. Mean age was 64.23 ± 9.59 (range 43–89) years in the 3D HUD group and 64.91 ± 9.67 (range 46–85) years in the SOM group (*p* = 0.70). The female: male ratio was 28:42 in the 3D HUD group and 16:54 in the SOM group (*p* = 0.05). In 55.7% (*n* = 39) of cases in the 3D HUD group and 62.9% (*n* = 44) of cases in the SOM group, vitrectomy was combined with cataract extraction and IOL implantation (*p* = 0.99). A total of 26 eyes (37.1%) in the 3D HUD group and 23 eyes (32.9%) in the SOM group were already pseudophakic at the time of retinal detachment surgery (*p* = 0.72). The mean postoperative follow-up including BCVA testing, slitlamp and fundus examination was 3.29 ± 2.29 (range 2–11) months in the 3D HUD group and 3.86 ± 4.29 (range 2–31) in the SOM group (*p* = 0.07). As this was a retrospective case series in a “real-world” tertiary care center setting, follow-up time was not perfectly consistent in all cases. However, the vast majority of patients (95.7%) were seen between 8 and 18 weeks postoperatively following retinal detachment surgery, resulting in a mean follow-up time of 3.6 months and a median of exactly 3 months for all patients (3D and SOM group). There was no statistically significant difference between groups with respect to preoperative BCVA, the extent of retinal detachment, the prevalence of macular detachment, the number of retinal tears as well as the occurrence of giant retinal tears.

**Table 1:** Groups' baseline characteristics.

	**3D (70 eyes)**		**SOM (70 eyes)**		
**Parameter**	**Mean ± SD**	**Range**	**Mean ± SD**	**Range**	***p*-value**
Female/male ratio	28/42		16/54		0.05
Age (years)	64.23 ± 9.59	43–89	64.91 ± 9.67	46–85	0.70
Right eye/left eye ratio	47/23		33/37		0.48
Pseudophakic eyes	26		23		0.72
extent of detachment (in quadrants)	2.60 ± 0.71	1–4	2.34 ± 0.72	1–4	0.07
Macula On/Off ratio	40/30		40/30		0.99
Giant retinal tear	14		13		0.99
Number of tears	3.33 ± 1.78	1–9	3.16 ± 1.91	1–9	0.40
BCVA preop (logMAR)	0.71 ± 0.72	0.00–2.40	0.75 ± 0.78	0–00–2.70	0.99

Main surgical outcomes are summarized in Table 2. Surgical time was significantly longer in the 3D group (3D HUD: 66.2 ± 16.5 min, range 36–113; SOM: 61.2 ± 17.1, range 34–115; *p* = 0.04). However, while this was apparent when looking at the first 35 cases (3D HUD: 65.29 ± 16.38 min, range 36–105; SOM: 58.83 ± 16.01 min, range 35–115; *p* = 0.03; Table 3), it was not reproducible when only comparing the latest 35 cases against each other (Table 4) therefore suggesting a learning curve for the 3D HUD (3D HUD: 67.2 ± 16.9 min, range 43–113; SOM: 63.5 ± 18.1 min, range 34–114; *p* = 0.49). No intraoperative complications were encountered in either group. The proportion of eyes that received a silicone oil tamponade was similar between groups (3D HUD: 10%, *n* = 7; SOM: 4.3%, *n* = 3; *p* = 0.32). In regard to functional outcome, the 3D HUD and SOM groups achieved comparable BCVA of 0.26 ± 0.40 (range 0.00–2.40) logMAR and 0.21 ± 0.38 (range 0.00–2.70) logMAR, respectively (*p* = 0.96). Concerning anatomical outcome, the rate of successful retinal reattachment was comparably high in the 3D HUD group (88.6%, 62/70) and the SOM group (94.3%, 66/70; *p* = 0.37). Postoperative PVR was noted in 12.6% (*n* = 9) of cases in the 3D HUD group and in 7.1% (*n* = 4) of cases in the SOM group (*p* = 0.40). Albeit the frequencies of retinal re-detachment and postoperative PVR showed no statistically significant difference, a trend toward more cases of retinal re-detachment and postoperative PVR might be seen in the 3D HUD group. This trend was neither statistically confirmed when looked at the last 35 cases individually (Table 4). The incidence of retinal re-detachment was found there to be identical in both groups (*n* = 2; *p* = 0.99). In the last 35 cases, 8.6% (*n* = 3) showed postoperative PVR in the 3D HUD group and 5.7% (*n* = 2) in the SOM group (*p* = 0.99; Table 4). In 3D HUD cases the trend goes down from 17.1% (*n* = 6) to 8.6% (*n* = 3; *p* = 0.48) for postoperative PVR and from 17.1% (*n* = 6) to 5.7% (*n* = 2) for retinal re-detachment (*p* = 0.26; Table 5). On the contrary, this trend was not apparent in the SOM group where the rate was identical for retinal re-detachment (*n* = 2; *p* = 0.99) and nearly identical for the incidence of postoperative PVR with 8.6% (*n* = 2) in the first 35 and 5.7% (*n* = 2) in the last 35 cases (*p* = 0.99; Table 6).

**Table 2:** Treatment parameters, anatomical and visual outcomes (all pars plana vitrectomies).

	**3D (70 eyes)**		**SOM (70 eyes)**		
**Parameter**	**Mean ± SD**	**Range**	**Mean ± SD**	**Range**	***p*-value**
Oil/Gas tamponade	7/63		3/67		0.32
Cataract extraction	39		44		0.99
Primary reattachment rate	62		66		0.37
Postoperative PVR	9		5		0.40
Length of operation	66.23 ± 16.53	36-113	61.19 ± 17.14	34-115	0.04
Postop macular edema	10		10		0.99
BCVA postop (logMAR)	0.26 ± 0.40	0.00-2.40	0.21 ± 0.38	0.00-2.70	0.96

**Table 3:** Comparison of the first 35 cases (3D HUD vs. SOM).

	**3D (35 eyes)**		**SOM (35 eyes)**		
**Parameter**	**Mean ± SD**	**Range**	**Mean ± SD**	**Range**	***p*-value**
Female/male ratio	19/16		7/28		0.28
Age (years)	65.26 ± 10.00	43–89	66.49 ± 10.97	46–85	0.63
Right eye/left eye ratio	26/9		12/23		0.09
Extent of detachment (in quadrants)	2.69 ± 0.72	1–4	2.43 ± 0.70	1–4	0.24
Macula “On”/“Off” - ratio	17/18		21/14		0.50
Giant retinal tear	7		6		0.99
Number of retinal tears	3.69 ± 1.94	1–9	2.94 ± 1.89	1–8	0.08
Oil/Gas	5/30		1/34		0.20
Cataract extraction	18		21		0.63
Primary reattachment rate	29		33		0.26
Postoperative PVR	6		3		0.50
Length of operation	65.29 ± 16.38	36–105	58.83 ± 16.01	35–115	0.03
Postop macular edema	3		4		0.99
BCVA preop (logMAR)	0.72 ± 0.61	0.00–2.40	0.78 ± 0.73	0.00–1.82	0.98
BCVA postop (logMAR)	0.37 ± 0.46	0.00–2.40	0.22 ± 0.46	0.00–2.70	0.24

**Table 4:** Comparison of the last 35 cases (3D HUD vs. SOM).

	**3D (35 eyes)**		**SOM (35 eyes)**		
**Parameter**	**Mean ± SD**	**Range**	**Mean ± SD**	**Range**	***p*-value**
Female/male ratio	9/26		9/26		0.99
age (years)	63.20 ± 9.18	46–81	63.34 ± 8.03	52–79	0.90
Right eye/left eye ratio	21/14		21/14		0.99
Extent of detachment (in quadrants)	2.51 ± 0.70	1–4	2.26 ± 0.74	1–4	0.67
Macula “On”/“Off” - ratio	23/12		19/16		0.44
Giant retinal tear	7	1–7	7		0.99
Number of retinal tears	3.00 ± 1.50		3.40 ± 1.88	1–9	0.46
Oil/Gas	2/33		2/33		0.99
Cataract extraction	21		23		0.80
Primary reattachment rate	33		33		0.99
Postoperative PVR	3		2		0.99
Length of operation	67.17 ± 16.87	43–113	63.54 ± 18.12	34–114	0.49
Postop macular edema	7		6		0.99
BCVA preop (logMAR)	0.71 ± 0.72	0.00–2.40	0.71 ± 0.75	0.00–1.2	0.93
BCVA postop (logMAR)	0.26 ± 0.40	0.00–2.40	0.21 ± 0.38	0.00–1.0	0.24

**Table 5:** Comparison of the first 35 cases and the last 35 cases operated in 3D.

	**3D (case 1–35)**		**3D (case 36–70)**		
**Parameter**	**Mean ± SD**	**Range**	**Mean ± SD**	**Range**	***p*-value**
Female/male ratio	19/16		9/26		0.04
age (years)	65.26 ± 10	43–89	63.20 ± 9.18	46–81	0.38
Right eye/left eye ratio	26/9	1–4	21/14	1–4	0.61
Extent of detachment (in quadrants)	2.69 ± 0.72		2.51 ± 0.70		0.45
Macula “On”/“Off” - ratio	17/18		23/12		0.22
Giant retinal tears	7		7		0.99
Number of retinal tears	3.69 ± 1.94	1–9	3.00 ± 1.50	1–7	0.14
Oil/Gas	5/30		2/33		0.42
Cataract extraction	18		21		0.64
Primary reattachment rate	29		33		0.26
Postoperative PVR	6		3		0.48
Length of operation	65.29 ± 16.38	36–105	67.17 ± 16.87	43–113	0.64
Postop macular edema	3		7		0.31
BCVA preop (logMAR)	0.72 ± 0.61	0.00–2.40	0.71 ± 0.72	0.00–2.40	0.30
BCVA postop (logMAR)	0.37 ± 0.46	0.00–2.40	0.26 ± 0.40	0.00–2.40	0.007

**Table 6:** Comparison of the first 35 cases and the last 35 cases operated with SOM.

	**SOM (case 1–35)**		**SOM (case 36–70)**		
**Parameter**	**Mean ± SD**	**Range**	**Mean ± SD**	**Range**	***p*-value**
Female/male ratio	7/28		9/26		0.99
age (years)	66.49 ± 10.97	46–85	63.34 ± 8.03	52–79	0.18
Right eye/left eye ratio	12/23	1–4	21/14	1–4	0.09
Extent of detachment (in quadrants)	2.43 ± 0.70		2.26 ± 0.74		0.99
Macula “On”/“Off” - ratio	21/14		19/16		0.82
Giant retinal tears	6		7		0.99
Number of retinal tears	2.94 ± 1.89	1–8	3.40 ± 1.88	1–9	0.21
Oil/Gas	1/34		2/33		0.99
Cataract extraction	21		23		0.80
Primary reattachment rate	33		33		0.99
Postoperative PVR	3		2		0.99
Length of operation	58.83 ± 16.01	35–115	63.54 ± 18.12	34–114	0.22
Postop macular edema	4		6		0.47
BCVA preop (logMAR)	0.78 ± 0.73	0.00–1.82	0.71 ± 0.75	0.00–1.20	0.42
BCVA postop (logMAR)	0.22 ± 0.46	0.00–2.70	0.21 ± 0.38	0.00–1.00	0.59

In the last 35 cases in the 3D HUD group, BCVA was significantly better (0.26 ± 0.4 vs. 0.37 ± 0.46 logMAR; *p* = 0.007; Table 5). Further characteristics and comparisons between the first and the last 35 cases are summarized in Tables 5, 6.

As the only postoperative complication encountered in either group, cystoid macular edema requiring topical or parabulbar corticosteroid therapy was noted in 14.3% (*n* = 10) of cases in both groups (*p* = 0.99). There were no occurences of endophthalmitis, vitreous hemorrhage or hypotony. As expected from the relatively short follow-up time (median 3 months), no cases of ERM were encountered through our oct scans in the presented series in either group. Further long term follow-up seems warranted to reveal any potential differences regarding ERM formation between groups.

## Discussion

The present study demonstrated comparable safety and efficacy between a 3D HUD setting and tradition SOM based vitreoretinal surgery for primary rhegmatogenous retinal detachment in a cohort of 140 eyes, offering non-inferior rates of retinal redetachment, postoperative PVR and final visual acuity. Interestingly, surgery time was slightly longer in the 3D HUD group. Our findings corroborate previous case reports and smaller case series indicating the potential feasibility of 3D HUD for posterior segment surgery (5, 6, 10–12).

Albeit not being statistically significant, the 3D HUD group showed a tendency toward a higher incidence for retinal re-detachment and postoperative PVR when comparing all 140 cases. This difference was however not observed when only comparing the last 70 cases against each other, which indicates that a 3D HUD setting might require time to adapt (“learning curve”). This notion seems confirmed by the fact that the final visual acuity outcomes in the 3D HUD group improved with the number of cases and was statistically significantly better in the last 35 than in the first 35 cases.

Coppola et al. (6) reported similar results in safety and efficacy for retinal detachment surgery although the smaller case series (*n* = 7 in the 3D group) and other missing variables could not provide a direct comparison but at least indicate a trend. On the contrary, Zhang Z. et al. (12) examined only complex cases for vitreoretinal detachment including cases with preoperative PVR, recurrent retinal detachment and cases with proliferative diabetic retinopathy (PDR). They found that, in general, 3D HUD required lower endoillumination levels for complex vitreoretinal surgery and provided the surgeon with improved ergonomics. However, some surgeons in this study experienced nausea and dizziness when performing laser coagulation and assistants were complaining of difficulty and fatigue when performing scleral indentation (12). Parameters for outcome and efficacy were not investigated or reported.

Assessing the clinical non-inferiority of 3D HUD as compared to SOM, Zhang T. et al. (11) compared 14 first time rhegmatogenous retinal detachment cases operated in 3D HUD vs. 34 retinal detachments operated with with a SOM setting. No statistical significant differences in postoperative BCVA and surgical time were observed here, albeit the numbers for retinal detachments operated in 3D might have been too little to draw a significant comparison.

In terms of general posterior segment surgery, Eckardt et al. provided a feasibility analysis using questionnaires to rate the surgeons' satisfaction in over 400 vitrectomies (including retinal detachment surgery). They found that—in clinical routine—the better ergonomics and the sharper image are just some factors that have been rated as major advantages (10). Other benefits specific to safety included that significantly fewer surgeron-related mistakes when performing specific manual exercises (such as placing sequins onto two needles located on a biconvex polystyrene disc) occurred using the 3D HUD system (10). In analogy, Zhang Z. et al. (12) found that both surgeons and residents expressed overwhelming preference for the 3D system, confirming the findings by Eckardt et al. on improved ergonomics.

On the contrary, the so-often reported “ease of use” was not confirmed by Talcott et al. (5), who reported a series of 39 peeling cases (including epiretinal membranes and macular holes). Additionally, their study reported a longer surgical time due to a longer peeling time which, similar to our study, indicates a learning curve for the 3D HUD. Surgeon satisfaction and ergonomics were not parameters that were tested in our study as it has been extensively studied in the past and our primary goal was to rate safety and efficacy of this system.

Other factors often investigated for posterior segment surgery with the 3D HUD system were endoillumination levels as retinal photoxicity from high endoillumination levels has previosuly been reported to be an issue for vitreoretinal surgery (13–17). Michels et al. for example observed postoperative macula lesions due to photic injury caused by high endoillumination levels in ocular surgery resulting in a decreased visual acuity outcomes. The authors then suggest carefully planning the surgery to decrease exposure time with high endoillumination levels (14). In addition to that, Koelbl et al. recently suggested that the allowed exposure times for illuminations systems are overestimated nowadays and that the intraocular irradiance is significantly higher than previously thought due to the spherical structure of the eye. This can cause the reflection of electromagnetic waves several times and lead to multiple intraocular light-tissue interactions reaching certain threshold values much quicker (17). For the 3D HUD in vitreoretinal surgery, most studies reported very low endoillumination levels, thus possibly reducing photoxity or photoxic stress on the retina (5, 10, 12, 18). Eventually, as investigated by Adam et al. (18), surgeons felt comfortable by going down to as low as 3% for vitrectomies.

Our study is mainly limited by its retrospective nature. Moreover, only primary (non-PVR-related) rhegmatogenous retinal detachment cases were included and further research is warranted to address the feasibility in more complex retinal detachment cases (e.g., proliferative diabetic retinopathy related tractional retinal detachment, or retinopathy of prematurity related retinal detachment). Another limitation is that the wide range of follow-up time might have influenced the rate of re-detachment recurrence. A strength of this study is its single surgeon design, thereby eliminating bias due to different experience levels. Another strength lies within the similarity between groups in regard to preoperative status, retinal detachment area, the rate of combination with cataract extraction, number of retinal tears, incidence of giant retinal tears and lastly the usage of gas and silicone oil tamponade.

To conclude, our investigation shows that using the 3D HUD system for vitrectomies on primary retinal reghmatogenous detachments can be used as a safe and efficient alternative to an SOM setting. While certain parameters as surgical time indicate a learning curve, these, and other trends were no longer present after the surgeon had grown familiar with the system. The advantages of 3D HUD (sharper image detail, better ergonomics, lower endoillumination levels) might be decisive in joining clinical routine in the future.

## Data Availability Statement

The original contributions generated for the study are included in the article/supplementary material, further inquiries can be directed to the corresponding author/s.

## Ethics Statement

Ethical review and approval was not required for the study on human participants in accordance with the local legislation and institutional requirements. The patients/participants provided their written informed consent to participate in this study.

## Author Contributions

BA: acquisition of data, analysis and interpretation of data, drafting of manuscript, review, and synthesis of literature. JS: conceptualization of the manuscript and review and synthesis of the literature, analysis and interpretation of data, critical review and revision of the manuscript. BS, TK, and WM: critical review and revision of the manuscript. NL and SP: conceptualization of the manuscript, critical review and revision of the manuscript, conception and design of the work/project, analysis and interpretation of data. All authors: contributed to the article and approved the submitted version.

## Conflict of Interest

JS: speaker honoraria and travel reimbursement from Carl Zeiss Meditec AG, Novartis Pharma GmbH, Bayer AG, Pharm-Allergan GmbH, Oculentis OSD Medical GmbH. Consultant: Bayer AG, Novartis Pharma GmbH, Pharm-Allergan GmbH. Travel reimbursement: Oertli AG. BS: speaker honoraria and travel reimbursement from Novartis Pharma GmbH and Topcon Corporation. WM: personal fees, travel reimbursement from Carl Zeiss Meditec, Alcon, Oculentis OSD Medical, Oertli AG, Staar Surgical, Pharm-Allergan, Ziemer, Ophthec, D.O.R.C, Bausch & Lomb. Advisory Board: Carl Zeiss Meditec, Alcon. TK: Speaker honoraria from Alcon Pharma GmbH. Personal consultation fees from Novartis Pharma GmbH and Bayer AG. Travel reimbursement from D.O.R.C. (International) B.V. NL: income from honoraria as a lecturer from Alcon Laboratories Inc., NIDEK Co., Ltd., and CenterVue SpA. SP: Speaker honoraria and travel reimbursement from Novartis Pharma GmbH, Oertli AG, Bayer AG, Alcon Pharma GmbH, Pharm-Allergan GmbH, Bausch & Lomb GmbH, Carl Zeiss Meditec AG.
